# Fractionated ^131^I anti-CEA radioimmunotherapy: effects on xenograft tumour growth and haematological toxicity in mice

**DOI:** 10.1038/sj.bjc.6604511

**Published:** 2008-08-05

**Authors:** J A Violet, J L J Dearling, A J Green, R H J Begent, R B Pedley

**Affiliations:** 1 Cancer Research UK Targeting and Imaging Group, Department of Oncology, University College London (Hampstead Campus), Rowland Hill Street, London NW3 2PF, UK

**Keywords:** radioimmunotherapy, anti-CEA antibody targeting, colorectal cancer, dose fractionation, ^131^I, CFU_gm_

## Abstract

Dose fractionation has been proposed as a method to improve the therapeutic ratio of radioimmunotherapy (RIT). This study compared a single administration of 7.4 MBq ^131^I-anti-CEA antibody given on day 1 with the same total activity given as fractionated treatment: 3.7 MBq (days 1 and 3), 2.4 MBq (days 1, 3, and 5) or 1.8 MBq (days 1, 3, 5, and 8). Studies in nude mice, bearing the human colorectal xenograft LS174T, showed that increasing the fractionation significantly reduced the efficacy of therapy. Fractionation was associated with a decrease in systemic toxicity as assessed by weight, but did not lead to any significant decrease in acute haematological toxicity. Similarly, no significant decrease in marrow toxicity, as assessed by colony-forming unit assays for granulocytes and macrophages (CFU_gm_), was seen. However, there was a significant depression of CFU_gm_ counts when all treated animals were compared with untreated controls, suggesting that treatment did suppress marrow function. In conclusion, in this tumour model system, fractionated RIT causes less systemic toxicity, but is also less effective at treating tumours.

Radioimmunotherapy (RIT) uses antibodies to deliver biologically targeted radiotherapy and has achieved considerable success in the treatment of the lymphomas and leukaemias (Press *et al*, 1995; DeNardo *et al*, 1999; Kaminski *et al*, 2000; Burke *et al*, 2002). However, success in the treatment of the common epithelial tumours has been less impressive. For example, in a recent review of RIT colorectal cancer looking at a large number of phase I/II clinical studies, only modest activity has been seen (Koppe *et al*, 2005). In external beam radiotherapy (EBRT), fractionation, dividing the total therapy dose into a number of smaller doses, is an established practise and reduces late normal tissue toxicity. This approach allows for significant increases in the total radiation dose that can be delivered, improving the therapeutic ratio (Hall, 1985). Another beneficial effect of fractionation is tumour shrinkage during therapy leading to a process of tumour revascularisation and re-oxygenation (Hall, 1985). Fractionation of RIT has been proposed as a method for improving its therapeutic ratio (DeNardo *et al*, 2002).

To build on earlier work, the purpose of this study was to investigate the therapeutic efficacy and toxicity of ^131^I-labelled A5B7, a monoclonal antibody binding to carcinoembryonic antigen (CEA), as either a single treatment or as part of a fractionated regimen in nude mice bearing CEA-expressing LS174T xenografts. A previous study using ^131^I-labelled A5B7 fragments did not demonstrate a therapeutic advantage to fractionation, although only a single fractionation regime (1 *vs* 3 doses) was investigated and no direct assessment of haematological toxicity was made (Pedley *et al*, 1993). In this study, a range of fractionation regimes have been investigated and a detailed assessment made of both systemic and haematological toxicities. The latter comprised measurement of both acute changes in mature blood indices and also an assessment of haematopoietic stem cell function.

## Materials and methods

### Antibody and radiolabeling

The monoclonal antibody A5B7 used in this study has been described previously (Rogers *et al*, 1984) and has been used pre-clinically and clinically for the RIT of CEA-expressing tumours (Begent *et al*, 1989; Pedley *et al*, 1991, 1993, 1994 and 2001; Lane *et al*, 1994). The antibody was radiolabelled on day 1 using the chloramine-T method (Greenwood and Hunter, 1963) at a ratio of 5 MBq ^131^I to 1 mg antibody and sterilised by passage through a 0.22 *μ*m acrodisc filter. Carcinoembryonic antigen binding was assessed on days 1 and 8 using a CEA-binding column, and stability of the radioimmunoconjugate over the course of the experiment was assessed using thin-layer chromatography. All experiments were in compliance with the UK Coordinating Committee on Cancer Research Guidelines for the Welfare of Animals in Experimental Neoplasia.

### Animal model

The human colorectal adenocarcinoma cell line LS174T grows as a moderate-to-poorly differentiated CEA-producing adenocarcinoma with small glandular acini (Tom *et al*, 1976), and was used to develop a tumour xenograft in the flanks of female MF1 nude (*nu/nu*) mice by subcutaneous implantation of small tumour pieces (∼1 mm^3^). At the initiation of the experiments, mice were aged 2–3 months and weighed 23–27 g. For all studies, the mice were given food and water *ad libitum*. Water contained 0.1% potassium iodide to block thyroid uptake of iodine.

### Biodistribution studies

Detailed biodistribution studies have previously been performed using ^131^I-A5B7 in both humans and this human xenograft model (Lane *et al*, 1994; Pedley *et al*, 1991, 2001). A limited biodistribution study was performed for this experiment before the therapy studies to confirm selective tumour localisation and retention, and also the absence of significant normal tissue uptake. Antibody biodistribution was assessed by administering 1.8 MBq of ^131^I-labelled radiolabelled antibody by tail vein injection into mice bearing LS174T xenografts (tumour volume was approximately 0.75 cm^3^). At 24 and 48 h, the animals (*n*=4 mice per group) were bled and liver, kidney, lung, muscle, colon, spleen, and tumour removed for comparative activity assessment using a LKB Wizard (Pharmacia, Milton Keynes, UK) gamma counter. Results are expressed as percentage injected dose per gram of tissue (% ID g^−1^).

### Therapy studies

To determine an appropriate fractionation schedule, a preliminary series of single therapy experiments were performed in tumour-bearing mice, with administered activities ranging from 1.8 to 11.1 MBq. After therapy, tumour growth and acute haematological toxicity were assessed as described above. On the basis of these studies, a total activity of 7.4 MBq was chosen for the fractionated study, as this was associated with significant growth delay and measurable white cell toxicity. Experiments commenced 7–10 days after passaging when the tumours were in exponential growth and had reached 0.1–0.2 cm^3^ in size. Groups of six mice received radioactivity delivered as a single administration of 7.4 MBq (day 1) or the same total activity divided into 2, 3, or 4 fractions of 3.7 MBq (days 1 and 3), 2.4 MBq (days 1, 3, and 5), or 1.8 MBq (days 1, 3, 5, and 8); control mice underwent tail vein injection of normal saline. Formulation of the fractionated activities was effected by diluting radiolabelled antibody made up on day 1 with normal saline for each treatment, taking into account of radioactive decay.

Tumours were measured every 2–3 days and mice were culled by cervical dislocation when the tumour volume reached 1.5 cm^3^. Tumour measurements were carried out in three dimensions (length, width, and height), and the tumour volume was estimated as length × width × height/2 (Looney *et al*, 1973).

### Toxicity studies

Therapy toxicity was assessed by a number of indices. Systemic toxicity was assessed by animal weights, with animals weighed on the day of antibody injection and every 2–3 days thereafter. Acute and late haematological toxicity was assessed using full blood counting and assays of early progenitor cell function. Mice underwent weekly removal of 25 *μ*l of whole blood for 1 month after the initiation of therapy. After dilution in physiological media, this blood was analysed using an automated haemocytometer (Advia 120, Bayer Diagnostics, Tarrytown, NY, USA) to give a haemoglobin level and white cell and platelet counts. Upon killing, marrow was taken from the mouse femurs to perform colony-forming unit assays for granulocytes and macrophages (CFU_gm_). Using aseptic technique, a superficial incision was made into the lower abdomen of the mice to pierce the skin. Subsequent removal of the skin of the lower limbs and a limited dissection of soft tissue from the femur was then performed to expose the knee and hip joints. An incision was then made through the proximal femur at the hip joint and just proximal to the knee. The marrow contents were flushed from the marrow cavity using 2 ml of phosphate-buffered saline and a 25-gauge needle. This cell suspension was then diluted in 3% acetic acid to lyse red blood cells and the mononuclear cells counted using a haemocytometer. Cells (10^5^) were mixed thoroughly with growth medium (Methocult™ CFU-GM, StemCell Technologies, London, UK), and incubated at 37°C for 7 days in a humidified chamber with 5% CO_2_. Colonies were counted using an inverted microscope with a similar method applied to age-matched controls.

### Statistics

Using the SPSS statistics package, the Kaplan–Meier analysis was used to assess the survival of the different treatment groups with a log-rank test to estimate any differences. Other intergroup analyses were performed using either a Mann–Whitney *U*-test to compare two groups or a one-way ANOVA where multiple comparisons were made.

## Results

### Radiopharmaceutical purity

There was a small increase in the proportion of free nuclide from less than 0.5% at day 1 to 5.9% at day 7.

### Antibody binding

Radiolabelled antibody was stored at 4°C in phosphate-buffered saline and antibody binding was assessed on days 1 and 7 using a CEA-binding column. This revealed a modest decrease in binding efficiency over the course of the experiment, falling from 86 to 70%. Radiopharmaceutical quality control was carried out by thin layer chromatography.

### Accuracy of repeated therapy administration

To check the accuracy of dilution and decay calculations, serial gamma counter measurements of radiation delivery were taken on each day of administration and revealed a high degree of consistency in actual administered activity for the fractionated therapies, with mean administered activity of 98.4% of baseline with a standard deviation of 3.5%.

### Biodistribution studies

**Figure 1:**
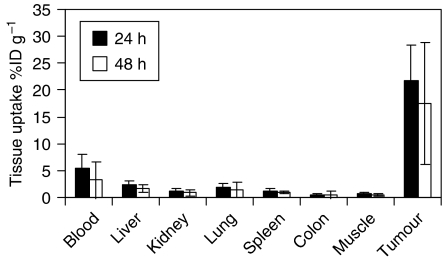
Distribution of ^131^I-labelled A5B7 at the 24 and 48 h time points (with associated standard deviations) in the tissues of mice bearing LS174T xenografts (*n*=4 at each time point).

Figure 1 shows the biodistribution of labelled antibody in groups of four mice given 1.8 MBq of ^131^I A5B7, which confirmed tumour localisation and clearance from normal tissue. At 24 h, 21.6% (±6.8) of injected activity was found in the tumour, whereas blood activity had already fallen to 5.3% (±2.7) and levels in other normal tissues were even lower. At 48 h, there was good retention of activity within tumour at 17.5% (±11.4) of injected activity, whereas blood activity had fallen further to 3.3% (±3.46).

### Therapy studies

**Figure 2:**
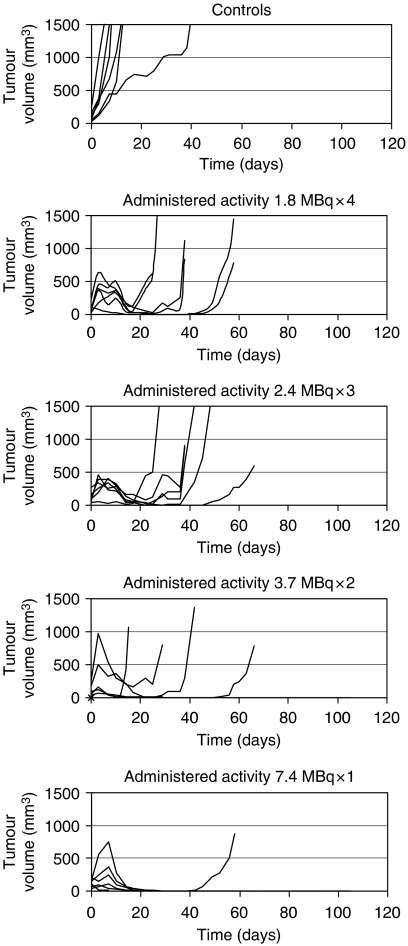
Individual tumour growth assessed using serial three-dimensional measurements of tumour size to estimate tumour volume.

**Figure 3:**
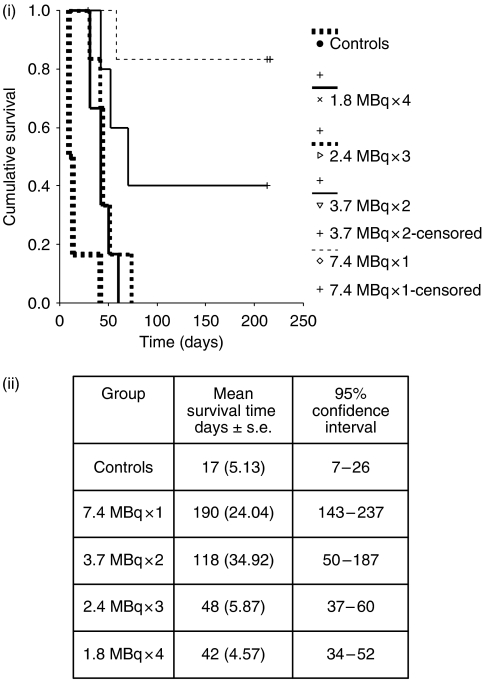
(i) The Kaplan–Meier plot showing the difference in survival of untreated and treated mice receiving 7.4 MBq of ^131^I-A5B7 administered as either a single therapy dose or multiple fractions. (ii) Mean survival times assessed by the log-rank method.

In the control group, exponential growth continued in all but one animal, whereas a short period of continued tumour growth was seen in all treated animals, followed by a reduction in tumour size before re-growth (Figure 2). The period of tumour growth inhibition was directly related to the activity of individual therapies, with cures seen in five of the mice receiving a dose of 1 × 7.4 MBq and two of the mice receiving 2 × 3.7 MBq, but none of the mice in the other treatment groups during the observation period of 230 days, at which point the experiment was terminated. The Kaplan–Meier method has been used to display the cumulative probability of mice surviving each of the treatment regimes and is shown in Figure 3i. The log rank was applied to test the null hypothesis that there was no difference in survival between the treatment groups and revealed that there were significant differences in survival between the different test groups. Group 1, receiving a single administration of 7.4 MBq (mean survival 190 days) had a significantly improved survival over all other treatment groups with the exception of the 3.7 MBq × 2 group (mean survival 118 days). No significant difference was found when the other treatment groups were compared with each other, but all the treatment groups had a significant survival advantage over the control group and are shown inFigure 3ii.

### Toxicity

#### Systemic toxicity: weight

**Figure 4:**
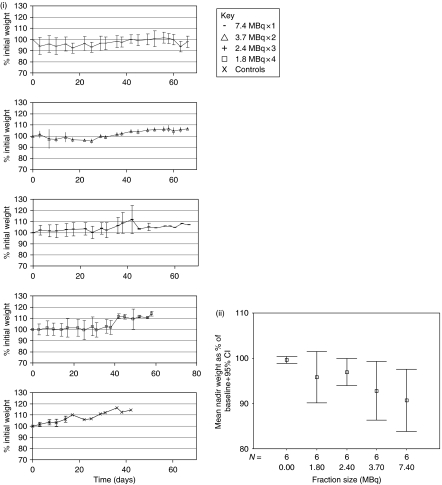
(i) Mean animal weights over time expressed as a percentage of baseline weight (±s.d.). (ii) Mean nadir weights as a percentage of baseline weight (±95% CI).

Control mice gained weight steadily during the course of the experiment, whereas weight gain was slower in all of the treatment groups (Figure 4i). A decrease of 10% in body mass was seen in two of the animals in the 7.4 MBq × 1 group, and a single animal in both the 3.7 MBq × 2 and 1.8 MBq × 4 groups. The mean nadir weight for each group is shown in Figure 4ii and analysis using a one-way ANOVA revealed a significant difference between the groups (*P*=0.033). *Ad hoc* testing demonstrated a significant decrease in body mass for the 7.4 MBq group compared with controls (*P*=0.039), suggesting increased systemic toxicity in this group.

#### Acute blood toxicity

**Figure 5:**
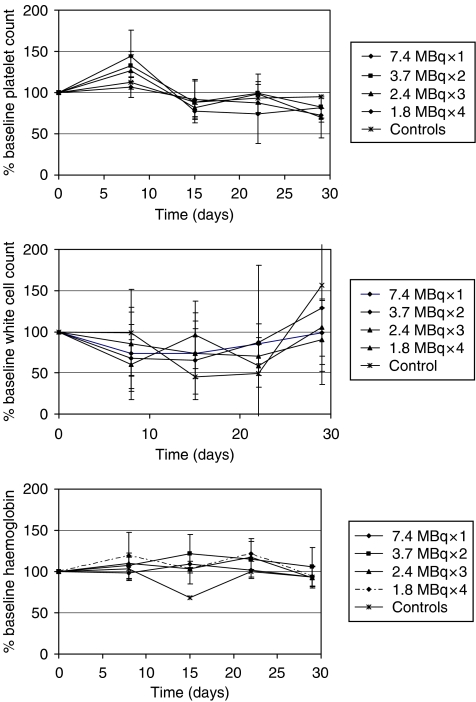
Blood indices as a percentage of baseline following therapy with associated standard deviations.

Owing to rapid tumour progression in the control group, only a single untreated control mouse was available for comparison with the therapy arms after day 8, and this data have therefore been excluded from the statistical analysis. Mean blood indices over time for each of the therapy groups are shown in Figure 5. To detect any differences between the blood counts as a result of therapy, the mean area under the curve (AUC) was calculated for each of the therapy groups and a one-way ANOVA was performed to look for differences between them. No significant differences between the mean AUC was found for haemoglobin (*P*=0.302), platelets (*P*=0.598), or total white cell count (*P*=0.763). Similarly, the acute nadir counts were also analysed in the same way and revealed no significant difference between any of the treatment groups for haemoglobin (*P*=0.688), platelets (*P*=0.256), or total white cell count (*P*=0.505).

#### Marrow progenitor cell toxicity

**Figure 6:**
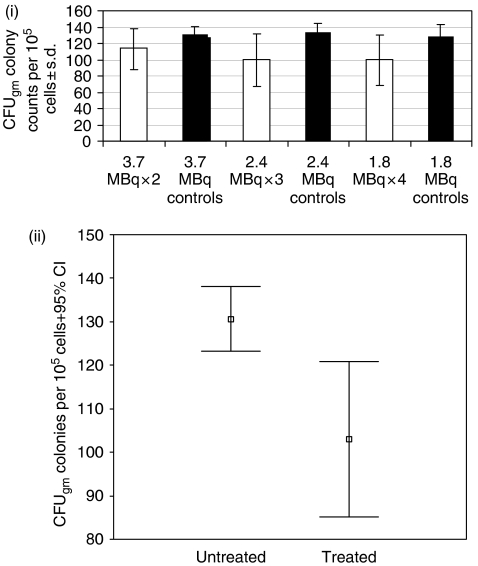
(i) Mean CFU_gm_ colony counts per 10^5^ mononuclear cells alongside those of age matched untreated controls +95% CI. (ii) Comparison between pooled CFU_gm_ colony counts per 10^5^ mononuclear cells for treated and untreated animals.

Femoral marrow harvesting was undertaken in therapy mice at the time of killing (with the exception of the control group) and also in age-matched controls, and used to perform CFU_gm_ or macrophages and granulocytes. Samples were tested for all the groups except for the 7.4 MBq group, where a number of samples failed as a result of technical problems. Colony counts per 10^5^ plated mononuclear cells are shown with their respective age-matched controls in Figure 6i. One-way ANOVA was used to look for differences in colony counts between any of the treatment or control groups and revealed no significant differences (*P*=0.795, 0.865, respectively). A Mann–Whitney *U*-test was also performed to analyse differences between the colony counts in each of the treatment groups and their respective age-matched controls and revealed no significant differences for the 2 × 3.7, 3 × 2.4 or 4 × 1.8 MBq groups (*P*=0.275, 0.513, and 0.275, respectively). Test data were subsequently pooled to look for any differences between the treated and non-treated animals and are shown in Figure 6ii. A statistically significant reduction in colony-forming units was found between the treated and untreated animals using both *t*-test and the Mann–Whitney *U*-test (*P*=0.005 and 0.011, respectively).

## Discussion

Owing to the high incidence of neutralising antibody formation seen using murine antibodies, most RIT to date has been performed using single therapy schedules. However, with the advent of chimeric and humanised antibodies of reduced immunogenicity, repeated therapy with RIT is now possible. Given the benefits seen from fractionation in EBRT, it has similarly been proposed as a method to improve the therapeutic ratio of RIT. However, a simple extrapolation from EBRT is not possible given that the radiobiology of daily bursts of high dose rate radiation, given over a number of weeks characteristic of EBRT, is different to that of RIT. Important key differences between RIT and EBRT include a lower total dose of radiation, a much lower dose rate of radiation delivery over prolonged periods of time, more akin to brachytherapy, and, finally, an exponentially decreasing dose rate.

In this study, using ^131^I-labelled A5B7 in nude mice bearing LS174T xenografts, the effects of fractionation of RIT have been investigated comparing tumour growth and toxicity. Previous work using the F(ab′)_2_ fragment of A5B7 in the same xenograft model system, comparing single administration of antibody with the administration of the same activity as three separate fractions, demonstrated reduced efficacy as a result of fractionation (Pedley *et al*, 1993). Similarly, in this study, fractionation appeared to have a clear detrimental effect upon long-term cure of tumours. Cures were seen in five out of six animals receiving the single fraction therapy and two out of six animals receiving a bi-fractionated administration, whereas no cures were seen in either the three or four fraction groups.

These findings are at odds to a number of preclinical studies that have suggested that fractionation of RIT is beneficial, with improvements in therapeutic effect even in the absence of dose escalation (Schlom *et al*, 1990; Buchsbaum *et al*, 1995; Goel *et al*, 2001; Bloechl *et al*, 2005a). In a recent study, looking at fractionated intraperitoneal RIT in a murine gastric cancer tumour model, a clear therapeutic advantage was seen following fractionation. In this study, which used a ^213^Bi-labelled antibody, the administration of two injections of antibody at a 7-day interval was more effective than a single injection of twice the activity (Bloechl *et al*, 2005b). The factors that may be responsible for this increase in therapeutic efficacy have been reviewed and include a significant ‘inverse dose rate’ effect, increased tumour shrinkage during therapy leading to improved tumour blood flow, a reduction in tumour interstitial pressure improving the access of subsequently therapy doses, and, in addition, an increased time for tumour re-oxygenation to occur (DeNardo *et al*, 2002). Pre-clinical studies have also demonstrated that fractionated RIT improves the delivery of antibody to the well vascularised, actively growing areas of tumour (Roberson *et al*, 1997; Buchsbaum *et al*, 1999).

One possible explanation for the discrepancy seen with some of the published data in this area is that the total dose of radiation delivered to tumour sites in our fractionated regime was less than that achieved by a single administration. Modest reductions in CEA binding of A5B7 over the course of therapy are evident by day 7 *in vitro* and may have reduced the efficiency of targeting, hence reducing the total dose. Furthermore, there were also modest falls in radiopharmaceutical purity, with a progressive decrease in the proportion of bound to free antibody over the course of administration, something that may also be expected to reduce the tumour-absorbed dose. However, the size of these changes appears modest in comparison with the difference in tumour control seen in the study. In addition, reduced vascular permeability as a result of prior radiation exposure has also been noted 7–21 days after RIT in other tumour model systems, and has been found to lead to reductions in tumour uptake of a second dose of radiolabelled antibody (Blumenthal *et al*, 1991, 1995). Although such changes are tumour-type dependent, the LS174T xenograft has demonstrated a reduced vascular permeability as a result of RIT (Blumenthal *et al*, 1997). Finally, conventional radiobiology and application of the linear quadratic model predict that radiation is less effective as the dose rate is lowered due to repair of sublethal damage requiring a higher total dose for similar effect (Dale, 1985, 1996; Langmuir and Sutherland, 1988; Fowler, 1990). In addition, the longer overall treatment times associated with fractionated RIT will allow greater tumour repopulation during therapy.

In terms of systemic toxicity, this appeared to be reduced as a result of fractionation. No significant reductions in acute haematological toxicity or CFU_gm_ counts were seen, although a nonsignificant trend towards lower CFU_gm_ counts in each of the therapy groups compared with their controls was seen, suggesting that the lack of significance reflected the relatively small number of data points. The therapy group data were therefore pooled and a subsequent comparison of treated and untreated animals did indeed demonstrate a significant depression of CFU_gm_ function as a result of therapy. The apparent lack of reduction in haematological toxicity by fractionation could be explained by the low rates of radiation delivery by RIT, meaning therapy is already highly fractionated and that any further benefits in terms of normal tissue sparing resulting from further fractionation are likely to be minimal (Dale, 1996). In addition, red marrow and its progenitor cells are known to behave as early responding tissues with limited repair capacity. As a result, fractionation might be expected to make little difference to toxicity (Hendry and Lajtha, 1972; Chu-Tse and Lajtha, 1975).

The findings in this study are similarly at odds with other pre-clinical studies using colorectal xenografts, which have suggested that fractionated approaches may be less myelotoxic than single therapy regimes (Schlom *et al*, 1990; Beaumier *et al*, 1991; Vriesendorp *et al*, 1993). In the study by Beaumier *et al* (1991), delayed and incomplete recovery of WBC counts was seen after single administration of therapy, whereas this was not seen after the administration of a multifraction regime of equivalent administered activity. In the study by Schlom *et al* (1990), marrow aplasia resulted from a single administration of radiolabelled antibody, whereas animals receiving the equivalent administered activity in a fractionated manner avoided this complication. However, the most comprehensive study of the effects of fractionated RIT on haematological toxicity has been undertaken in non-tumour-bearing animals (Vriesendorp *et al*, 1993). Acute haematological toxicity was assessed by daily blood counts, and an assessment of late marrow toxicity made by assaying early marrow progenitor cells using CFU_gm_ assays. They found that single injections of RIT were associated with earlier and more severe haematological toxicity (thrombocytopenia and granulocytopenia) and lower bone marrow CFU_gm_ counts. However, an important difference between this study and other pre-clinical studies is that the majority of these have myeloablative activities of radiation, and then derived fractionated therapy schedules from this. Published data on the behaviour of marrow progenitor cells have shown that they have a high ability to repopulate as long as marrow ablation does not occur (Hendry and Lajtha, 1972; Testa *et al*, 1974; Chu-Tse and Lajtha, 1975; Hendry and Lord, 1983), and the total dose of radiation used in this study was not myeloablative when given as a single dose.

## Conclusions

In this tumour model system, for a fixed administered activity, fractionating RIT reduced efficacy. Fractionation was not associated with any reduction in either acute blood toxicity or early marrow progenitor cell function, although the latter was reduced in treated animals compared with controls. However, systemic toxicity, as assessed by weight, was reduced.

## References

[bib1] Beaumier PL, Venkatesan P, Vanderheyden JL, Burgua WD, Kunz LL, Fritzberg AR, Abrams PG, Morgan ACJ (1991) 186Re radioimmunotherapy of small cell lung carcinoma xenografts in nude mice. Cancer Res 51: 676–681 1845957

[bib2] Begent RH, Ledermann JA, Green AJ, Bagshawe KD, Riggs SJ, Searle F, Keep PA, Adam T, Dale RG, Glaser MG (1989) Antibody distribution and dosimetry in patients receiving radiolabelled antibody therapy for colorectal cancer. Br J Cancer 60: 406–412 2789951 10.1038/bjc.1989.295 PMC2247181

[bib3] Bloechl S, Beck R, Seidl C, Morgenstern A, Schwaiger M, Senekowitsch-Schmidtke R (2005a) Fractionated locoregional low-dose radioimmunotherapy improves survival in a mouse model of diffuse-type gastric cancer using a 213Bi-conjugated monoclonal antibody. Clin Cancer Res 11(19 Part 2): 7070s–7074s 16203804 10.1158/1078-0432.CCR-1004-0017

[bib4] Bloechl S, Beck R, Seidl C, Morgenstern A, Schwaiger M, Senekowitsch-Schmidtke R (2005b) Fractionated locoregional low-dose radioimmunotherapy improves survival in a mouse model of diffuse-type gastric cancer using a 213Bi-conjugated monoclonal antibody. Clin Cancer Res 11(19 Part 2): 7070s–7074s 16203804 10.1158/1078-0432.CCR-1004-0017

[bib5] Blumenthal RD, Kashi R, Sharkey RM, Goldenberg DM (1995) Quantitative and qualitative effects of experimental radioimmunotherapy on tumor vascular permeability. Int J Cancer 61: 557–566 7759161 10.1002/ijc.2910610421

[bib6] Blumenthal RD, Sharkey RM, Kashi R, Goldenberg DM (1991) Suppression of tumor vascular activity by radioantibody therapy: implications for multiple cycle treatments. Sel Cancer Ther 7: 9–16 1925118 10.1089/sct.1991.7.9

[bib7] Blumenthal RD, Sharkey RM, Kashi R, Sides K, Stein R, Goldenberg DM (1997) Changes in tumor vascular permeability in response to experimental radioimmunotherapy: a comparative study of 11 xenografts. Tumour Biol 18: 367–377 9372870 10.1159/000218051

[bib8] Buchsbaum DJ, Khazaeli MB, Liu TP, Bright S (1995) Fractionated radioimmunotherapy of human colon cancer xenografts with I-131 labelled monoclonal antibody CC49. Cancer Res 55(Suppl): 5881–5887 7493364

[bib9] Buchsbaum DJ, Khazaeli MB, Mayo MS, Roberson PL (1999) Comparison of multiple bolus and continuous injections of ^131^I-labeled CC49 for therapy in a colon cancer xenograft model. Clin Cancer Res 5(10 Suppl): 3153s–3159s 10541357

[bib10] Burke JM, Jurcic JG, Scheinberg DA (2002) Radioimmunotherapy for acute leukemia. (Review) (42 refs). Cancer Control 9: 106–113 11965231 10.1177/107327480200900203

[bib11] Chu-Tse W, Lajtha LG (1975) Haemopoietic stem-cell kinetics during continuous irradiation. Int J Radiat Biol Relat Stud Phys Chem Med 27: 41–50 1078817 10.1080/09553007514550041

[bib12] Dale RG (1996) Dose-rate effects in targeted radiotherapy. (Review) (46 refs). Phys Med Biol 41: 1871–1884 8912367 10.1088/0031-9155/41/10/001

[bib13] Dale RG (1985) The application of the linear-quadratic dose effect equation to fractionated and protracted radiotherapy. Br J Radiol 58: 515–528 4063711 10.1259/0007-1285-58-690-515

[bib14] DeNardo GL, Schlom J, Buchsbaum DJ, Meredith RF, O'Donoghue JA, Sgouros G, Humm JL, DeNardo SJ (2002) Rationales, evidence, and design considerations for fractionated radioimmunotherapy. Cancer 94(4 Suppl): 1332–1348 11877764 10.1002/cncr.10304

[bib15] DeNardo SJ, DeNardo GL, Kukis DL, Shen S, Kroger LA, DeNardo DA, Goldstein DS, Mirick GR, Salako Q, Mausner LF, Srivastava SC, Meares CF (1999) 67Cu-2IT-BAT-Lym-1 pharmacokinetics, radiation dosimetry, toxicity and tumor regression in patients with lymphoma. J Nucl Med 40: 302–310 10025839

[bib16] Fowler JF (1990) Radiobiological aspects of low dose rates in radioimmunotherapy. Int J Radiat Oncol Biol Phys 18: 1261–1269 2347734 10.1016/0360-3016(90)90467-x

[bib17] Goel A, Augustine S, Baranowska-Kortylewicz J, Colcher D, Booth BJ, Pavlinkova G, Tempero M, Batra SK (2001) Single-dose *vs* fractionated radioimmunotherapy of human colon carcinoma xenografts using ^131^I-labeled multivalent CC49 single-chain fvs. Clin Cancer Res 7: 175–184 11205906

[bib18] Greenwood FC, Hunter WM (1963) The Preparation of 131I-Labelled human growth hormone of high specific radioactivity. Biochem J 89: 116–123 10.1042/bj0890114 PMC1202279 14097352

[bib19] Hall EJ (1985) Radiation biology. Cancer 55: 2051–2057 3919919 10.1002/1097-0142(19850501)55:9+<2051::aid-cncr2820551404>3.0.co;2-y

[bib20] Hendry JH, Lajtha LG (1972) The response of hemopoietic colony-forming units to repeated doses of X-rays. Radiat Res 52: 309–315 4643160

[bib21] Hendry JH, Lord BI (1983) The analysis of the early and late response to cytotoxic insults in the haematopoetic cell hierarchy. In: Cytotoxic Injury to Tissue, Potten CS, Hendry JH (eds), Churchill Livingstone: Edinburgh, UK, p 1

[bib22] Kaminski MS, Estes J, Zasadny KR, Francis IR, Ross CW, Tuck M, Regan D, Fisher S, Gutierrez J, Kroll S, Stagg R, Tidmarsh G, Wahl RL (2000) Radioimmunotherapy with iodine (131)I tositumomab for relapsed or refractory B-cell non-Hodgkin lymphoma: updated results and long-term follow-up of the University of Michigan experience. Blood 96: 1259–1266 10942366

[bib23] Koppe MJ, Bleichrodt RP, Oyen WJ, Boerman OC (2005) Radioimmunotherapy and colorectal cancer. (Review) (86 refs). Br J Surg 92: 264–276 15739250 10.1002/bjs.4936

[bib24] Lane DM, Eagle KF, Begent RH, Hope-Stone LD, Green AJ, Casey JL, Keep PA, Kelly AM, Ledermann JA, Glaser MG (1994) Radioimmunotherapy of metastatic colorectal tumours with iodine-131-labelled antibody to carcinoembryonic antigen: phase I/II study with comparative biodistribution of intact and F(ab′)2 antibodies. Br J Cancer 70: 521–525 8080740 10.1038/bjc.1994.338 PMC2033373

[bib25] Langmuir VK, Sutherland RM (1988) Radiobiology of radioimmunotherapy. Antibody, Immunoconjugate, Radiopharmaceutical 1: 195–211

[bib26] Looney WB, Mayo AA, Allen PM, Morrow JY, Morris HP (1973) A mathematical evaluation of tumour growth curves in rapid, intermediate and slow growing rat hepatomata. Br J Cancer 27: 341–344 4349700 10.1038/bjc.1973.41 PMC2008786

[bib27] Pedley RB, Begent RH, Boden JA, Boden R, Adam T, Bagshawe KD (1991) The effect of radiosensitizers on radio-immunotherapy, using 131I-labelled anti-CEA antibodies in a human colonic xenograft model. Int J Cancer 47: 597–602 1995488 10.1002/ijc.2910470420

[bib28] Pedley RB, Begent RH, Boden JA, Boxer GM, Boden R, Keep PA (1994) Enhancement of radioimmunotherapy by drugs modifying tumour blood flow in a colonic xenograft model. Int J Cancer 57: 830–835 8206678 10.1002/ijc.2910570611

[bib29] Pedley RB, Boden JA, Boden R, Dale R, Begent RH (1993) Comparative radioimmunotherapy using intact or F(ab′)2 fragments of 131I anti-CEA antibody in a colonic xenograft model. Br J Cancer 68: 69–73 8318423 10.1038/bjc.1993.288 PMC1968289

[bib30] Pedley RB, Hill SA, Boxer GM, Flynn AA, Boden R, Watson R, Dearling J, Chaplin DJ, Begent RH (2001) Eradication of colorectal xenografts by combined radioimmunotherapy and combretastatin a-4 3-*O*-phosphate. Cancer Res 61: 4716–4722 11406542

[bib31] Press OW, Eary JF, Appelbaum FR, Martin PJ, Nelp WB, Glenn S, Fisher DR, Porter B, Matthews DC, Gooley T (1995) Phase II trial of 131I-B1 (anti-CD20) antibody therapy with autologous stem cell transplantation for relapsed B cell lymphomas. Lancet 346: 336–340 7623531 10.1016/s0140-6736(95)92225-3

[bib32] Roberson PL, Dudek S, Buchsbaum DJ (1997) Dosimetric comparison of bolus and continuous injections of CC49 monoclonal antibody in a colon cancer xenograft model. Cancer 80: 2567–2575 9406711 10.1002/(sici)1097-0142(19971215)80:12+<2567::aid-cncr32>3.3.co;2-4

[bib33] Rogers GT, Rawlins GA, Kardana A, Gibbons AR, Bagshawe KD (1984) A monoclonal antibody against a CEA-related antigen expressed on HT29 colon tumour cells. Eur J Cancer Clin Oncol 20: 1279–1286 6208034 10.1016/0277-5379(84)90257-8

[bib34] Schlom J, Molinolo A, Simpson JF, Siler K, Roselli M, Hinkle G, Houchens DP, Colcher D (1990) Advantage of dose fractionation in monoclonal antibody-targeted radioimmunotherapy. J Natl Cancer Inst 82: 763–771 2182892 10.1093/jnci/82.9.763

[bib35] Testa NG, Hendry JH, Lajtha LG (1974) The response of mouse haematopoetic colony-forming units to repeated whole-body X irradiation. Biomedicine (Express) 21: 431 4464991

[bib36] Tom BH, Rutzky LP, Jakstys MM, Oyasu R, Kaye CI, Kahan BD (1976) Human colonic adenocarcinoma cells. I. Establishment and description of a new line. In Vitro 12: 180–191 1262041 10.1007/BF02796440

[bib37] Vriesendorp HM, Shao Y, Blum JE, Quadri SM, Williams JR (1993) Fractionated intravenous administration of 90Y-labeled B72.3 GYK-DTPA immunoconjugate in beagle dogs. Nucl Med Biol 20: 571–578 8358342 10.1016/0969-8051(93)90025-p

